# Ginsenoside Rg3: Potential Molecular Targets and Therapeutic Indication in Metastatic Breast Cancer

**DOI:** 10.3390/medicines6010017

**Published:** 2019-01-23

**Authors:** Maryam Nakhjavani, Jennifer E Hardingham, Helen M Palethorpe, Yoko Tomita, Eric Smith, Tim J Price, Amanda R Townsend

**Affiliations:** 1 Molecular Oncology, Basil Hetzel Institute, The Queen Elizabeth Hospital, Woodville South, SA 5011, Australia; maryam.nakhjavani@adelaide.edu.au (M.N.); helen.palethorpe@adelaide.edu.au (H.M.P.); yoko.tomita@adelaide.edu.au (Y.T.); eric.smith@adelaide.edu.au (E.S.); 2 Adelaide Medical School, University of Adelaide, Adelaide, SA 5005, Australia; timothy.price@sa.gov.au (T.J.P.); amanda.townsend@sa.gov.au (A.R.T.); 3 Oncology Unit, The Queen Elizabeth Hospital, Woodville South, SA 5011, Australia

**Keywords:** Ginsenoside Rg3, breast cancer, AQP-1, epimer, angiogenesis

## Abstract

Breast cancer is still one of the most prevalent cancers and a leading cause of cancer death worldwide. The key challenge with cancer treatment is the choice of the best therapeutic agents with the least possible toxicities on the patient. Recently, attention has been drawn to herbal compounds, in particular ginsenosides, extracted from the root of the Ginseng plant. In various studies, significant anti-cancer properties of ginsenosides have been reported in different cancers. The mode of action of ginsenoside Rg3 (Rg3) in in vitro and in vivo breast cancer models and its value as an anti-cancer treatment for breast cancer will be reviewed.

## 1. Metastatic Breast Cancer

**Figure 1:**
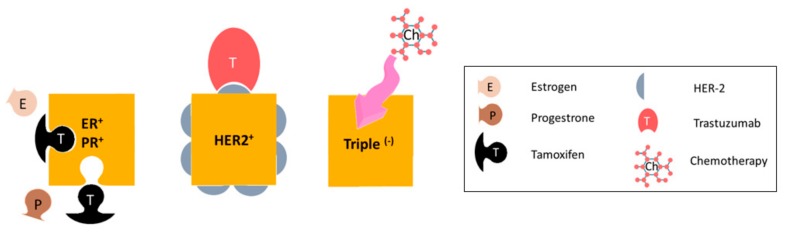
Subtypes of metastatic breast cancer, based on receptor expression. Hormone-receptor expressing tumors are treated with anti-hormone therapy (such as tamoxifen), HER2-expressing tumors are given targeted anti-HER2 monoclonal antibody therapy such as trastuzimab. The main treatment option for triple negative breast tumors is chemotherapy.

Metastatic breast cancer (MBC) is classified as stage IV where the tumor has metastasized to distant organs such as bones, lungs, liver, or brain [1]. Breast tumors are subdivided into different categories based on the receptors expressed in the cells: estrogen receptor (ER)- or progesterone receptor (PR)- positive, human epidermal growth factor receptor 2 (HER2) positive, and triple negative breast tumors (ER^-^/PR^-^/HER2^-^) (Figure 1). Hormone receptor expressing tumors including luminal A or luminal B subtypes constitute the largest portion of patients and have the best prognosis, as these tumors are inherently less aggressive compared to other subtypes of breast cancer and that the majority of these tumors are responsive to hormone therapy options such as tamoxifen or letrozole [2]. HER2-expressing tumors constitute 15–20% of the patients [3]. The prognosis of this group of patients has improved following the introduction of targeted anti-HER2 medications, such as trastuzumab [4]. Some 15% of patients have triple negative (basal-like) breast cancer (TNBC) [5] and for these patients, chemotherapy remains the main treatment option [6]. TNBC is associated with younger age (<50 years) at diagnosis and BRCA1 mutation, and by the time of diagnosis have larger tumor size, higher grade tumor and the worst prognosis [7]. Recently, inhibitors of poly ADP ribose polymerase (PARP) have been found to be effective in the treatment of TNBC patients with BRCA-1 or -2 mutations. Likewise, platinum-based cytotoxic drugs are being tested in such patients [2]. However, still there is no ultimate cure for MBC and these patients suffer from the side effects of chemotherapy, until the tumor develops acquired resistance [8] and the patients succumb to the disease. Hence, many cancer researchers are actively looking for better treatment options to improve the quality of life of the MBC patients, reduce the toxicities of the chemotherapy regimens, restrict tumor metastasis, and improve survival. In this review, the medicinal herb ginseng and the important group of chemicals in its extract are suggested as one such therapy with the potential for reduced toxicity.

## 2. Ginseng—History and Medicinal Use

Ginseng, with a long history of human use as a traditional medicine, has various pharmacological effects [9,10,11], and is widely used for its nutritional value as a food, energizing the body [10], improving body performance in sports, relieving menopausal symptoms, and alleviating sexual dysfunction [12]. *Panax ginseng*, also known as Chinese or Korean ginseng is a species of ginseng, the extract of which has the highest medicinal value among other species. Ginsenosides are the group of chemicals in this extract having the highest medicinal value [13]. So far, 38 ginsenosides have been identified [14]. Ginsenosides are saponins having a steroid-like hydrophobic backbone connected to sugar moieties. Based on their chemical structure, they are categorized into panaxadiol, panaxatriol, and oleanolic groups [13], with protopanaxadiols being the most abundant group. Protopanaxadiols include Rb1, Rb2, Rg3, Rh2, Rc, Rd, Ra, and F2 [12] and have various medicinal properties, including anti-diabetic effects, protection against cardiovascular diseases and anticancer properties [10,12]. 

Not all of these protopanaxadiols are available in ginseng extract. Commercial ginseng is produced either by air-drying or steaming (120°C, 4 h) the plant. These processes produce white or red ginseng, respectively [15,16,17]. Due to the conversions and chemical changes following heating, the heat processed or red ginseng has higher medicinal properties than white ginseng. Within this heating process, polar ginsenosides such as Rb1 or Rb3 convert to less polar ginsenosides such as Rg2 and Rg3 [15,18]. 

**Figure 2:**
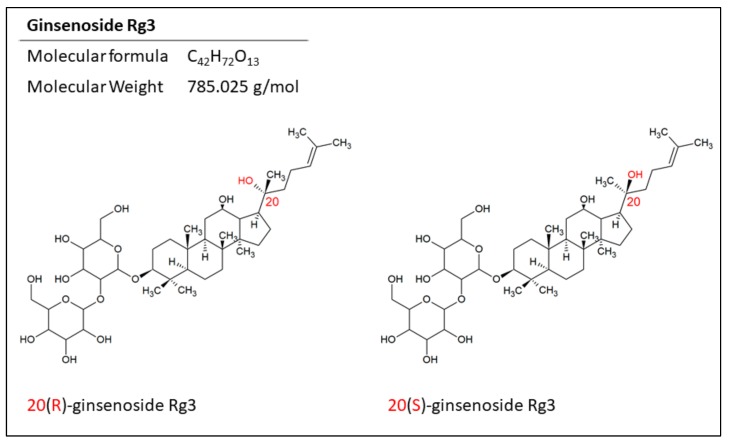
The structure of the epimers of ginsenoside Rg3. The position of the hydrogen on C_20_ makes two epimers for this molecule.

Ginsenoside Rg3 is one of the well-studied members of protopanaxadiols, and arises following loss of the sugar moiety on C_20_ in the heating process. Like other ginsenosides, Rg3 has a stereocenter on C_20_, giving it two epimers; 20(R)- and 20(S)-ginsenoside Rg3 (Figure 2). This is due to the selective attachment of the hydroxyl group to the C_20_ after losing the sugar structure. In the 20(R)-Rg3 epimer, the hydroxyl on C_20_ is far from the hydroxyl on C_12_, whilst in 20(S)-Rg3, these two hydroxyls are close to each other. Also, the alkene chain connected to C_20_ in 20(R)-Rg3 is more flexible compared to its fixed orientation in 20(S)-Rg3. This makes the alkene in 20(S)-Rg3 less accessible to water and more prone to hydrophobic interactions while leaving the hydroxyl groups to interact with the receptors. This may play a part in the increased water solubility of 20(S)-Rg3 compared with 20(R)-Rg3 [19,20].

## 3. Epimers of Ginsenoside Rg3 in the Treatment of Cancer

Depending on the biological system being tested, the two epimers of ginsenoside Rg3, 20(S)- and 20(R)-Rg3, have distinct effects. For example, while both epimers inhibited the 5-HT_3A_ and α3β4 nACh receptors, only 20(S)-Rg3 inhibited the voltage-dependent Ca^2+^, K^+^, and Na^+^ channel currents [21,22]. The 20(S)-Rg3 was also a better scavenger of hydroxyl radicals [23] and in the human gastric cancer cell line AGS, was responsible for inducing apoptosis (through activation of caspase-3, -8, and -9) [24]. In human hepatocellular carcinoma cell line HepG2, 20(S)-Rg3 was the more effective epimer in inhibiting cell growth, downregulating the expression of DNA methyltransferases, reducing global DNA methylation, and in particular, modifying the methylation of the promoter region of some relevant genes in cancer such as VEGF, TP53, and BCL-2 [25]. 

In contrast, 20(R)-Rg3 was a better antioxidant against the oxidative stress induced by cyclophosphamide in mice [26] and can promote the immune response in mice better than 20(S)-Rg3 [27,28]. It was also a better inhibitor of tumor growth in mice bearing H22-transplanted hepatocellular tumors [28]. Epimers of Rg3 have also been tested in epithelial–mesenchymal transition (EMT) in lung adenocarcinoma in vitro models. For instance, 20(R)-Rg3 epimer inhibited EMT via increasing the expression of E-cadherin and inhibiting the expression of vimentin and upregulation of Snail [29].

## 4. Mechanisms of Action of Ginsenoside Rg3 in Breast Cancer

Regardless of the stereotype, Rg3 has been studied in several cancer models and various mechanisms are suggested for its actions. These mechanisms include induction of apoptosis [24,30,31,32,33,34,35,36,37,38,39,40,41,42,43,44,45,46,47,48,49,50,51,52], induction of autophagy through upregulation of autophagy-associated molecules [53], inhibition of proliferation [24,25,38,41,42,44,45,46,50,51,54,55,56,57,58,59,60,61,62,63], inhibition of metastasis [29,50,62,64,65,66,67,68,69,70,71] and angiogenesis [55,56,66], cell cycle arrest [47], immunomodulatory effects [72], sensitization to radiation [73], reducing multidrug resistance [74], and inducing genotoxicity to the cancer cells [75]. A few studies have focused on the effects of Rg3 in breast cancer models; these mechanisms are discussed as follows.

### 4.1. Induction of Apoptosis and Inhibition of Proliferation

Induction of apoptosis is one of the most studied mechanisms of action of Rg3 in different cancers. Apoptosis is a complex process, regulated by extrinsic (via the death receptor) and intrinsic (via mitochondrial) pathways. The intrinsic pathway is activated by DNA damage and oxidative stress whilst the extrinsic pathway can be triggered by the activation of the members of the TNF receptor superfamily. Both pathways ultimately activate caspase enzymes. 

**Figure 3:**
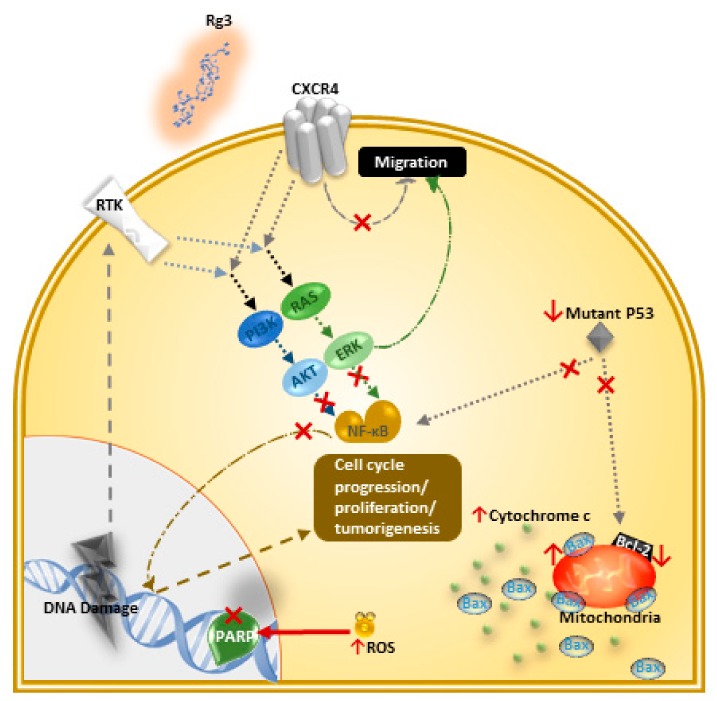
Rg3 inhibits cell proliferation and induces apoptosis via different effector molecules and pathways, in MDA-MB-231 cell line [30,31]. Changes in specific molecules involved in signalling pathways upon exposure of the cells to Rg3 is shown in this figure. The **↑** and **↓** arrows are indicating increased and decreased levels of certain molecules, respectively, and the **×** signs show the inhibition of a signalling pathway or function of a certain protein in the MDA-MB-231 cell line.

Caspases can interact with an apoptosis inhibitor such as inhibitors of apoptosis proteins (IAP) and the Bcl-2 family. Caspases can also go through auto-activation and cleave other substrates, one of which is PARP, an important DNA repair enzyme. In a TNBC cell line, MDA-MB-231, Rg3 activated caspase-3, and degraded PARP through the generation of reactive oxygen species (ROS) [30]. In addition, Rg3 caused an increased ratio of pro-apoptotic Bax and the anti-apoptotic Bcl-2 [30]. Also, it inhibited the binding of NF-κB to DNA. NF-κB is a transcription factor that is constitutively active in breast cancer cells and drives further cell cycle progression, proliferation and inhibition of apoptosis. Proteins Akt and ERK are two kinases involved in the activation of NF-κB and it is observed that in MDA-MB-231 cell line, Rg3 inhibited the phosphorylation of Akt and ERK and hence prevented the activation of NF-Κb [31]. P53, a tumor suppressor protein, has a negative regulatory effect on Bcl-2 while mutant P53 can prolong the activation of NF-κB and affect the apoptosis of cancer cells. In MDA-MB-231 cells, Rg3 destabilized mutant P53, suppressed the expression of Bcl-2, and induced apoptosis [31]. Figure 3 summarizes these mechanisms. 

Inhibition of proliferation is another important function proposed for Rg3. For example, in in vivo settings, Rg3 has shown inhibition of tumor growth in the cancer models of colon [54], lung [38,72], liver [39], pancreas [76], and gallbladder [32]. As a specific epimer, 20(S)-Rg3 has caused similar responses in tumors of the gallbladder [47] and ovary [51,68] and 20(R)-Rg3 in melanoma [61,63], lung [62], and liver [28] cancer models. In vitro, in MCF-7 breast cancer cell lines, 20(S)-Rg3 (100-300 µM), caused a cell cycle arrest in G_1_-phase and hence inhibited cell proliferation [57]. Table 1 shows other suggested mechanisms of Rg3 in induction of apoptosis and inhibition of proliferation in other cancer models.

### 4.2. Inhibition of Migration, Invasion, Angiogenesis, and Metastasis

Ginsenoside Rg3 has been shown to reduce the migration, invasion, and angiogenesis of human umbilical vein endothelial cells (HUVECs) both in vitro and in vivo. Treatment of HUVECs with 20(R)-Rg3 reduced cell viability with an IC_50_ of 10 nM. There was a dose-dependent reduction in the tube forming capacity of these cells (1-1000 nM) and inhibition of VEGF-induced chemo-invasion in vitro. In vivo Rg3 inhibited angiogenesis (150 and 600 nM) in a matrigel plug assay. The mechanisms suggested were inhibition of matrix metalloproteinase (MMP)-2 and -9 [77]. 

Rg3 has been shown to degrade serum levels of IGF-1 and hence inhibits angiogenesis and tumor growth in breast cancer [78]. In a study by Chen et al. [69], in the MDA-MB-231 cell line, 20(S)-Rg3 decreased the expression of CXCR4, an important chemokine receptor expressed by breast cancer cells which is involved in migration and invasion (Figure 3) [69]. Other suggested mechanisms for this action in other cancer models include decreased expression of MMP-2 [29,77], -9 [66], and -13 [67], reducing the expression of HIF-1α [55,68], AQP1 [71], and HDAC3 [63], suppressing NF-κB and its products (c-Myc, COX-2, MMP-9) [55,64], inhibiting TGF-β1, inactivating proteins involved in EMT (p38 MAPK and Smad2) [29,55], downregulating FUT4 and EGFR mediated migration through MAPK and NF-κB [55,62], and decreasing the expression of VEGF [55] and VEGF dependent p38/ERK signaling [56] (Table 2). Rg3 has also resulted in an increased survival in mice bearing melanoma [50] and liver tumors [39].

### 4.3. Multidrug Resistance (MDR) and Combination Therapy

Rg3 can decrease MDR via inducing membrane fluidity and blocking drug efflux in leukemia cell lines [74]. In the Caco-2 cell line, 20(S)-Rg3 (80 µM) was shown to inhibit P-glycoprotein (Pgp) [79]. It has also been shown to increase the accumulation of drugs such as vincristine in MDR cells, but not in sensitive cells [80]. Likewise, mice bearing MDR tumors showed an increased survival time and less tumor weight when treated with a combination of doxorubicin and Rg3, rather than doxorubicin alone [80]. 

Few studies have shown the effects of co-administration of Rg3 and a chemotherapy agent in breast tumor models. What is known so far is that mice bearing breast tumors that received a combination of continuous low-dose capecitabine, a prodrug of fluorouracil (5-FU), and Rg3 showed less toxicity induced by capecitabine, longer survival, and reduced susceptibility to drug resistance [81]. This is in part due to the antiangiogenic effect of Rg3 as evidenced by the decreased VEGF expression and reduced microvasculature density. The outcomes of this study are promising for the oral administration of capecitabine and improvement in tolerance of the patients.

In addition, Rg3 when co-administered orally with paclitaxel, significantly increased the relative bioavailability of paclitaxel and decreased the relative breast tumor growth rate in mice bearing MCF-7 xenograft [79]. Rg3 has been tested in other cancer models in combination with cyclophosphamide [82,83], gemcitabine [84], temozolomide [85], cisplatin [86,87,88], docetaxel [89,90], doxorubicin [91,92], and As_2_O_3_ [93] (Table 3). 

### 4.4. Aquaporin (AQP) 1—a Putative Target of Rg3

**Figure 4:**
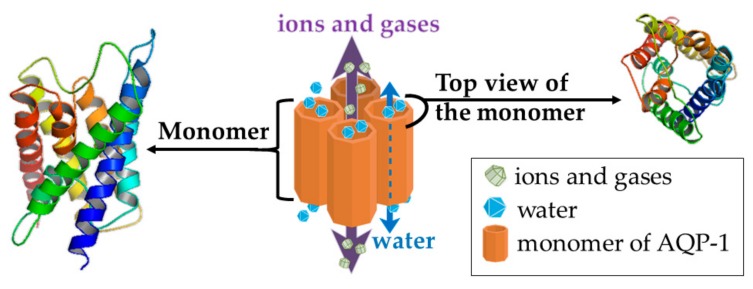
The structure of AQP1 channel, as a homotetramer, with the dashed arrow showing the water passage through the water channel of each monomer. The solid violet arrow represents the passage of ions and gases. The 3D structures were prepared in PyMol, version 1.7.4.5 (Schrödinger, Inc, Tokyo, Japan).

One suggested mechanism of action of Rg3 is by targeting AQP1 [71]. This molecule has roles in tumor growth, angiogenesis [94,95,96], metastasis [97,98], acquired resistance in tumors [99], and is highly expressed in aggressive tumors [100]. AQP1 is a member of the family of AQP membrane channels which are primarily known for their role in water transport across the lipophilic cell membrane. AQP1 was the first of the 13 members of the AQP proteins to be discovered [95,101,102]. It is a unique AQP in that, as well as acting as a water channel, it has a second function of transporting single charged cations, regulated by cGMP gating. AQP1 also transports gases such as nitric oxide, carbon dioxide, and ammonia (Figure 4) [102,103,104,105]. Rg3 was found to inhibit expression of AQP1 at both the mRNA and protein levels. Further, in a prostate cancer cell line (PC-3M), Rg3 (up to 10 µM) did not affect cell proliferation but inhibited cell migration in a transwell assay. Overexpression of AQP1 in this cell line attenuated the effect of Rg3 in inhibiting migration while silencing AQP1 gene via shRNA resulted in reduced PC-3M cell migration, and a diminished response to Rg3. These results indicated a critical role for AQP1 mediating the anti-migratory role of Rg3 [71]. This suggests that Rg3 targeting AQP1 could also be relevant in breast tumors.

#### AQP1 and Breast Cancer

In vitro data suggest that stable overexpression of AQP1 in MCF-7 (ER^+^ and PR^+^) and MDA-MB-231 (TNBC) breast cancer cell lines significantly increases cell invasion and proliferation [106]. In HUVECs, expression of AQP1 is known to be upregulated by estrogen, because the promoter of the AQP1 gene has a functional estrogen response element (ERE) and the homodimerized complex of estrogen-ER can activate this ERE [107]. AQP1 is expressed in all microvasculature endothelial cells including HUVECs. In HUVECs, estrogen increased the proliferation, migration, invasion, and tube forming capacity, and these effects were inhibited by knockdown of AQP1 expression using siRNA [107]. Epidermal growth factor stimulation induced translocation of AQP1 from the cytoplasm to the cell membrane to enhance cell invasion [106]. AQP1 was also found to colocalize with ezrin, a cytoskeletal protein involved in the proliferation, cell adhesion, and NO production in the endothelial cells [107].

Animal studies show that AQP1 is highly expressed in mouse breast tumor [108], and AQP1-null mice show impaired angiogenesis [109]. In mouse models of breast carcinoma with lung metastasis, AQP1 deficiency decreased the expression of VEGFR2 leading to significantly reduced tumor mass and volume, microvasculature density, and the number of lung metastases [110]. 

In humans, AQP1 is abundantly expressed in the endothelium of many tissues [111] including the endothelium of tumor micro-vasculature, positive for CD31 [100,107]. In normal breast tissues, the expression of AQP1 is low and limited to the ducts, lymphatics and connective tissue microvessels [112]. Breast cancer is one of the tumors with increased microvessels and angiogenesis compared to its matched normal tissue and there is an increased AQP1 expression in the microvasculature of breast tumor [112,113]. Breast tumors of basal-like TNBC subtype and advanced breast tumors have higher levels of AQP1 expression compared to normal tissues [112,113,114]. Benign breast lesions and ductal carcinoma in situ samples express AQP1 on the membrane of myoepithelial cells of the ducts, but the majority of invasive ductal carcinoma samples predominantly express AQP1 in the cytoplasm [106,115]. So far, studies have shown that membrane AQP1 expression is associated with triple-negativity, expression of cytokeratin 14 and smooth muscle actin, higher tumor grade, medullary-like histology and poor clinical prognosis [113,114]. High AQP1 expression was found to be an independent prognostic factor in the high-grade subgroup, in the ER-negative subgroup and in the node-negative subgroup [114]. Cytoplasmic expression of APQ1 is correlated with lymph node metastasis and advanced features of invasive ductal carcinoma [106]. 

### 4.5. Other Suggested Mechanisms of Action

Rg3, when orally administered to mice bearing lung tumors, had immunomodulatory effects, causing increased splenocyte proliferation [72]. It also increased genotoxicity in osteosarcoma cell lines, through increased DNA damage and double-strand breaks [75]. This compound sensitized lung cancer tumors to radiation via suppressing the activation of NF-κB and the proteins regulated by this transcription factor (such as COX2, MMP-9, VEGF, c-Myc, and cyclin D1), which are either induced by radiation or are involved in radio-resistance [73]. 

## 5. Metabolism and Pharmacokinetics of Rg3

Together with the clinical trials, it is pertinent to consider the pharmacokinetics of Rg3 following oral administration. So far, various studies have focused on the metabolism and pharmacokinetics of Rg3, as a general compound, and 20(R)-Rg3 in in vitro, animal models and healthy human volunteers. It is not yet clarified whether the metabolism of 20(S)- and 20(R)-Rg3 differ in any aspects. The general understanding is that following oral administration, ginsenosides undergo a partial or complete hydrolysis in the acidic conditions of the stomach and the intestinal microbial flora [116,117]. Rg3, like other protopanaxadiol ginsenosides, can lose a sugar moiety following metabolism by the anaerobic intestinal bacteria [118]. Compound K, the final metabolite of the metabolism of ginsenosides can be detected in human plasma after seven hours post-ingestion [118].

In vitro studies have shown that Rg3 has interactions with isoenzymes of cytochrome P450. Rg3 can weakly inhibit CYP3A4, moderately inhibit CYP2C19 and CYP1A2, and potently inhibit CYP2D4 [119] and so interactions between Rg3 and the drugs that are mainly metabolized with these isoenzymes should be considered. Also, incubation of Rg3 with human fecal microflora resulted in the formation of ginsenoside Rh2 [120], another member of the ginsenoside family with anticancer properties [121]. Studies in dogs however failed to detect any Rh2 in the plasma samples following oral or intravenous (IV) administration of 20(R)-Rg3 [122]. Although in vitro studies suggest that deglycosylation is one of the main pathways of the metabolism of Rg3, this study failed to show existence of such molecules in dog plasma samples [122]. This study also suggested a low degree of metabolism of 20(R)-Rg3, as evidenced by the maximum of 70% of 20(R)-Rg3 recovered from bile [122]. 

In rats, deglycosylation and oxygenation are reported as two major routes of metabolism for 20(R)-Rg3 [123]. The half-life of Rg3 in rats after an intravenous administration is reported to be 14 min [124] and 18.5 min [123]. The difference between these two reports might be due to the difference in the solubilisation of Rg3, however, both suggest a rapid rate of metabolic clearance for this molecule. Absolute bioavailablility of Rg3 in rats was about 2.63% [125].

Intra-species differences seem to play an important role in the metabolism and pharmacokinetics of Rg3, since in healthy human volunteers, Rg3 can be detected in the plasma for 8 [126,127] and up to 216 hours [126], following oral and intramuscular (IM) administration, respectively.

The epimers of Rg3 also differ in terms of tissue distribution. 20(S)-Rg3, following oral administration of 68 mg/kg to Sprague–Dawley rats was more concentrated in the gastrointestinal tissues compared to the plasma. It was also highly distributed in the liver, with the concentration being four and three times the plasma concentration at two and four hours, respectively. The concentration of Rg3 in other tissues such as muscle, spleen, lung, and fat was similar or lower than plasma concentration and trace amounts were detected in the brain, heart, and kidney. However, 20(R)-Rg3 was only localized in liver and the gastrointestinal tract, and not detected in the plasma [128]. Table 4 summarizes the results of the studies on the pharmacokinetics of Rg3 in animal models and human trials.

## 6. Clinical Trials

### 6.1. Application and Safety of Ginseng Extract on Healthy Human Volunteers

Studies on healthy human volunteers suggest that administration of the total extract of *Panax ginseng* C.A. Meyer is well tolerated and does not cause serious adverse reactions [130,131]. In a randomized, double-blind, placebo-controlled trial investigating the anti-oxidant properties of the total extract, 82 healthy volunteers received either placebo (n = 27), 1 or 2 g/day (n = 27 and n = 28, respectively) for a month [130]. Of the 82 volunteers, 80 completed the trial; only two, both female, randomized to receive 2 g/day of the total extract withdrew, one due to insomnia and palpitations after seven days, and the other due to non-health related reasons. Administration of the total extract improved the serum levels of anti-oxidant markers. In another randomized, double-blind, placebo-controlled trial investigating the anti-oxidant effects in postmenopausal women, 41 volunteers received placebo and 41 received 1 g of the extract thrice daily for 12 weeks [131]. Five volunteers receiving placebo and six receiving the extract failed to complete the trial. Administration of the total extract increased the enzyme activity of the serum antioxidant, superoxide dismutase, suggesting that the total extract may reduce oxidative stress in postmenopausal women. At this dose, reported side effects included dizziness, sleeplessness, nervousness, and uterine bleeding [131]. Adverse effects following administration of 1 or 2 g/day of total extract of ginseng to healthy subjects for a month were reported to be mild (constipation and dyspepsia, insomnia, and hot flash) and it was concluded that this extract does not cause serious adverse reactions and is safe and tolerable [132].

### 6.2. Clinial Trials and Application of Rg3 in Cancer Patients

Presently, there are only three published clinical trials utilizing Rg3 in the treatment of cancer; two on non-small cell lung carcinoma (NSCLC) [95] [96] and one on hepatocellular carcinoma (HCC) [99]. In the first study, a total of 133 patients with stage II-III NSCLC received either Rg3 alone (43 cases), Rg3 + chemotherapy (46 cases), or chemotherapy alone (44 cases) [133]. Rg3 was administered twice a day (0.8 mg/kg, equivalent to 40–50 mg/day) for at least 6 months. This study showed that Rg3 + chemotherapy improved the 3-year survival rates compared to either Rg3 or chemotherapy alone (54.3% versus either 46.5% or 47.7%, respectively; *p* > 0.05). In patients expressing VEGF, chemotherapy treatment alone resulted in decreased 3-year survival rates compared to patients with negative VEGF expression (*p* < 0.01); however, there were no significant differences for the other two groups. In addition, patients that received Rg3 had a lower incidence of adverse effects and better immune system function, as evidenced by the increased activity of NK cells and CD4^+^ T cells and the normal ratio of CD4^+^/CD8^+^ T cells [133]. This suggests that the option of combining Rg3 therapy with immunotherapy would be worth investigating.

In the second study, 124 patients with advanced (stage III-IV), unresectable NSCLC with EGFR mutations were divided into two groups receiving a tyrosine kinase inhibitor (TKI) + Rg3 (20 mg orally for at least 2 months) or TKI alone [134]. The results of this study demonstrated that Rg3 improved the median progression-free survival by 2.5 months (*p* = 0.049). Rg3 delayed the acquired resistance to TKI and had a low toxicity profile, with rash being the worst side effect in both groups and nausea, diarrhea, and anorexia being the most common side effects in both groups [134].

In the third study, 228 patients diagnosed with advanced (Barcelona clinic liver cancer-stage C) HCC were randomized in two groups, to receive trans-arterial chemoembolization (TACE) alone or in combination with Rg3 (20 mg, twice a day, orally) [135]. TACE is a successful method for delivering chemotherapy directly to the tumor within the liver which prolongs patient survival, but its application is limited by high recurrence rate, in part due to inflammatory factors promoting metastasis of the tumor. Inflammation and angiogenesis are associated phenomena in that pro-inflammatory cytokines such as IL-1ß or TNF-α released from activated neutrophils and macrophages cause vasculature modifications, enhancing proliferation of endothelial cells and hyper-neovascularization [136,137]. Hence, using an anti-angiogenic drug should limit this adverse effect. This study showed that the patients receiving TACE + Rg3 had longer median overall survival compared to those who received TACE alone (13.2 versus 10 months; *p* = 0.002), while there was no significant difference in progression free survival. Rg3 was well-tolerated, the reported adverse effects being grade 1 or 2 constipation, epistaxis, and hypertension, and importantly, Rg3 treatment tended to alleviate adverse effects related to TACE [135].

## 7. Conclusions

So far, many studies have shown the effects of Rg3 in different cancer models with fewer studies in human clinical trials. A limited number of studies have focused on the effects of Rg3 in breast cancer models and more specifically in advanced breast cancer. Out of the six studies on the effects of Rg3 in breast cancer, only one study has focused on the effects of an isomer, 20(S)-Rg3, and its in vivo effects in mice in increasing the efficacy of oral paclitaxel [79]. The rest of these studies used Rg3 as a whole compound [30,31,69,81,138]. In some cases, the source of the Rg3 used is self-produced, and of unknown purity [30,31]. Given the fact that Rg3 can have stereospecific activities [24,28,29], a mixture of two enantiomers, with unknown ratios of each enantiomer, cannot scientifically justify the resulting effects. With this view, stereospecific activity of Rg3 in human breast cancer models, including cell lines and patient tumor-derived cancer cells, in 2D and 3D in vitro models, and in vivo, is not known. Furthermore, considering the importance of AQP1 in angiogenesis and the invasiveness of tumors, together with evidence of survival benefit from clinical trials, the selectivity of Rg3 in targeting AQP1 in metastatic breast tumors should be studied. 

## Figures and Tables

**Table 1:** Suggested mechanisms for induction of apoptosis (IA) and inhibition of proliferation (IP) by Rg3 in various cancers are summarized in Table 1. The function of different epimers are indicated by symbols; * and ^◊^ represent 20(S)- and 20(R)-Rg3, respectively.

Cancer		Mechanism of Action	Reference
Ovary	IA	Downregulation of PI3K/Akt and the proteins of the IAP family * Activation of caspases -3 and -9 *	[52]
		Inhibition of Warburg effect by inactivation of Stat3 *	[51]
	IP	Suppression of the Warburg effect and modulating the Stat3/HK2 pathway	[51]
Colon	IA	Activation of AMPK * Increased DNA fragmentation, cleavage of PARP * Downregulation of Bcl-2 * Upregulation of p53, Bax, release of cytochrome c and caspase-3 and -9 *	[45,46]
	IP	Inhibiting the function of β-catenin and the ß-catenin/Tcf signalling Inhibits cell proliferation	[54]
		Reduced mitosis-related proteins * Reduced DNA-repair proteins *	[46]
		Changes in the Eph/ephrin signalling axis *	[58]
Lung	IA	Activation of the intrinsic and extrinsic pathways Regulation of apoptosis-associated proteins such as BCL2, BAX, PARP-1 Cleaving caspase-3 Inhibition of EGFR, Stat3, Akt and PI3/Akt signalling	[38,40,41]
	IP	Decreasing the expression of FUT4 and biosynthesis of LeY ^◊^ Decreasing the activation of EGFR and its downstream signaling ^◊^	[38,62]
		Suppression of some of the cell cycle proteins such as cyclin D1 and E, CDK-2 and -4 Suppression of some of the MAPK-associated growth proteins such as JNK, ERK and P38	[41]
Liver	IA	Activation of the intrinsic and extrinsic pathways through increasing Bax, caspase-3, release of cytochrome c, decreasing Bcl-2, Bcl-xL	[39,44,49]
		Sensitizing liver cancer cells to TRAIL-induced cell death Promoting TRAIL-induced caspase-dependent apoptosis (via DR5 upregulation and induction of CHOP)	[43]
Multiple myeloma	IA	Increasing the activity of caspase-3 and expression of Bax	[35]
	IP	Inhibiting the secretion of IGF-1 Affecting the Akt/mTOR signalling and their proliferation	[42]
Leukaemia	IA	Activating caspases -3 and -9 Downregulating PI3K/Akt family proteins	[48]
Gallbladder	IA	Increasing caspase-12 (an endoplasmic reticulum stress-mediated apoptosis)	[32]
		Activating p53 pathway and intrinsic apoptosis pathway * Inducing cell senescence *	[47]
Gastric	IA	Blocking TRMP7 Upregulation of caspase-3, -8, -9, Bax and downregulation of Bcl2	[24,34]
		Inhibiting the expression of FUT4 (via regulation of SP1 and HSD1) Activation of caspase-3, -8 and -9	[24,33]
Melanoma	IA	Preventing the binding of NF-κB to the FUT4 promoter Activating intrinsic and extrinsic apoptosis pathways	[36]
		Increasing the expression of caspase and Bcl-2 *	[50]
	IP	Decreasing the levels of active Akt * Dysregulating the PI3K/Akt pathway, hence affecting the cell cycle *	[50]
		Inducing a G0/G1 cell cycle arrest ^◊^ Decreasing the HDAC3 ^◊^ Increasing the acetylation and stability of p53 ^◊^	[63]
		Reducing FUT4 and LeY ^◊^ Inhibiting the EGFR/MAPK signalling pathway ^◊^	[61]
Glioblastoma multiforme	IA	Suppressing the MEK/MAPK signalling pathway and activating ROS by the antioxidant enzyme system, leading to apoptosis	[37]
Prostate	IP	Inhibition of DNA synthesis * Affecting the MAPK activity through ERKs, p38 and JNK *	[60]
Glioma	IP	Activating Akt and p53/p21 dependent signalling pathways causing cell senescence *	[59]

**Table 2:** Suggested mechanisms of inhibition of migration and invasion in different cancer models. The function of different epimers are indicated by symbols; * and ^◊^ represent 20(S)- and 20(R)-Rg3, respectively.

Cancer	Mechanism	Reference
Ovary	Inhibition of angiogenesis and cell invasion Decreased expression of MMP-9	[66]
	Blocking the EMT * Reducing HIF-1α expression *	[68]
Colon	Suppressing NF-κB and its products (c-Myc, COX-2, MMP-9)	[64]
Prostate	Decreasing the expression of AQP1 *	[71]
Melanoma	Inhibiting the expression of MMP-13 Reducing cell adhesion, invasion and angiogenesis *	[50,67,70]
	Decreasing the expression of HDAC3 ^◊^	[63]
Lung	Inhibiting TGF-β1 Inactivating proteins involved in EMT (MMP-2, p38 MAPK and Smad2) ^◊^	[29]
	Downregulating FUT4 and EGFR mediated migration (through MAPK and NF-κB) ^◊^	[62]
Endothelial progenitor cells	Decreasing the activation of the VEGF dependent p38/ERK signalling	[56]
Esophageal and renal	Decreasing the expression of VEGF Inhibiting other signalling pathways of HIF-1α, COX-2, NF-κB, STAT3 and MAPKs	[55]

**Table 3:** Suggested effects of Rg3 in combination with chemotherapy agents in in vitro and in vivo models.

Studied Model	Drug Combination	Effects	Reference
Lewis lung cancer mouse model	Rg3 + cyclophosphamide (continuous low-dose)	Less toxicity induced by capecitabine Longer animal survival Reduced susceptibility to drug resistance Increased anti-angiogenic activity	[82]
Mouse model	20(S)-Rg3 + cyclophosphamide	Inhibiting cyclophosphamide-induced DNA damages in the peripheral lymphocyte cells and bone marrow cells Reducing number of apoptotic cells of mice and improving the anti-oxidative markers in mice (such as SOD, MDA and GPX)	[83]
Mouse bearing hepatocellular carcinoma model	Rg3 + cyclophosphamide	Alteration of the expression of Bcl-2 family and induction of intrinsic pathway of apoptosis Prolonging mouse survival	[39]
Mouse bearing lung tumor model	Rg3 + gemcitabin	Enhancing the efficacy of gemcitabine on suppressing tumor growth Increasing the quality of life Prolonging mice survival Increasing tumor’s necrosis rate Decreasing VEGF expression, microvessel density (assessed by the expression of CD31) and arterial blood flow in tumors such as peak systolic velocity	[84]
Glioma cell line	Rg3 + temozolomide	Inducing cell cycle arrest and apoptosis Attenuating the expression of VEGF-a and Bcl-2	[85]
Glioma allograft model of mouse	Rg3 + temozolomide	Antiangiogenic effect (reduced relative cerebral blood volume, VEGF levels and microvessel density) Improving the antiangiogenic effects of temozolomide No additive effect on tumor growth	[85]
Mouse bearing colon tumor	Rg3 + cisplatin	Improving anti-cancer effects of cisplatin Inhibiting tumor growth Reducing the toxicities of cisplatin (decreasing the intracellular levels of ROS)	[86]
Kidney, liver and colon resistant cancer cells	Rg3 + cisplatin	Decreasing the high levels of etoxifying enzymes such as heme-oxygenase (HO-1) and NAD(P)H quinone oxidoreductase (NQO-1)	[86]
Cisplatin-resistant bladder tumor cell lines	Rg3 + cisplatin	Synergistic effect in inhibiting the proliferation (possibly through activating the intrinsic apoptosis pathway (decreased Bcl-2 and increased cytochrome c and caspase-3) and cell cycle alterations in G2/M phase)	[87]
Mouse bearing oesophageal squamous cell carcinoma	Rg3 + cisplatin	Enhancing the inhibitory effects of cisplatin Reducing the proliferation of cancer cells Decreasing the microvascular density of the tumors	[88]
Colon cancer cell lines	Rg3 + docetaxel	Sensitizing the cells to the docetaxel Improving its apoptotic effect (via inhibiting NF-κB and the expression of anti-apoptotic proteins such as Bcl-2, XIAP, and ciap-1) Increasing the expression of pro-apoptotic proteins (such as Bax, caspase-3 and -9)	[89]
Prostate cancer cell lines	Rg3 + docetaxel	Inhibiting cell growth Inducing apoptosis and its associated protein Arresting the cells at G0/G1 Modulating cell cycle-associated proteins Inhibiting the activity of NF-κB	[90]
Prostate cancer cell lines	Rg3 + docetaxel + cisplatin	More effective inhibition of the activity of NF-κB and cell growth	[90]
Mouse bearing hepatocellular tumor	20(S)-Rg3 + doxorubicin	Suppressing the autophagy via regulating autophagy-associated proteins Inhibiting autophagic flux Synergistic effects in inhibiting tumor growth	[91]
Rat model	Rg3 + doxorubicin	Reducing doxorubicin-induced cardiotoxicity (by improving the ejection fraction, fractional shortening and left ventricular outflow) Improving the oxidative damage and apoptosis induced by doxorubicin (via the activation of Akt and the Nrf2-ARE pathway)	[92]
NCI-H1299 lung cancer cells	Rg3 + As2O3	Inhibiting the proliferation of NCI-H1299 lung cancer cells	[93]
Mouse bearing lung tumors	Rg3 + As2O3	Promoting apoptosis in tumor cells Prolonging the survival of the mice	[93]

**Table 4:** A summary of the studies on the pharmacokinetics of Rg3 and the 20(R) epimer in in vivo models.

Ginsenoside	Model	Route	Dose	Sample	Detected Rg3	Outcomes	Reference
Rg3	Sprague–Dawley rats	IV	1 mg/kg	Plasma	Detected for 12 h	t_1/2α_: 0.12 ± 0.03 h	[125]
						t_1/2β_: 2.09 ± 0.50 h	
		Oral	10 mg/kg	Plasma	Detected for 12 h		
	Healthy humans	Oral	3.2 mg/kg	Plasma	Detected for 8 h	C_max_: 15.67 ± 6.14 ng/mL t_max_: 0.66 ± 0.01 h	[129]
	Healthy humans	IM	10, 30 and 60 mg	Plasma	Detected for 216 h		[126]
20(R)-Rg3	Sprague–Dawley rats	IV	5 mg/kg	Plasma	Detected for 1.5 h	t_1/2_: 14 min	[124]
				Urine	Not detected		
		Oral	50 mg/kg	Urine	Not detected in 1 h	rapid GI metabolism	
				Plasma	Not detected in 1 h		
	Dogs	IV	0.3 mg/kg	Plasma	Detected for 12 h	t_1/2_: 1.71 (± 0.11) h	[122]
		Oral	2 mg/kg	Plasma	Detected for 24 h	t_1/2_: 5.99 (± 1.16) h	
	Sprague–Dawley rats	IV	5 mg/kg, within 1 min	Plasma	Detected for 1.5 h	t_1/2_: 18.5 min	[123]
				Urine	Not detectable	N/A	
		Oral	100 mg/kg	Plasma	Not detectable	N/A	
				Urine	Not detectable		
				Feces	6 different deglycosylated and oxygenated metabolites		
	Healthy humans	Oral	3.2 mg/kg	Plasma	Detected for 8 h	t_max_: 0.66 ± 0.10 h C_max_: 1± 6 ng/mL t_1/2α_: 0.46 ± 0.12 h t_1/2β_: 4.9 1.1 h t_1/2(Ka)_: 0.28 ± 0.04 h	[127]
